# Cellular Protein Phosphatase 2A Regulates Cell Survival Mechanisms in Influenza A Virus Infection

**DOI:** 10.3390/ijms222011164

**Published:** 2021-10-16

**Authors:** Vanessa Gerlt, Juliane Mayr, Juliana Del Sarto, Stephan Ludwig, Yvonne Boergeling

**Affiliations:** 1 Institute of Virology Muenster, University of Muenster, 48149 Muenster, Germany; vanessa_gerlt@web.de (V.G.); mayrj@uni-muenster.de (J.M.); juliana.delsarto@ukmuenster.de (J.D.S.); ludwigs@uni-muenster.de (S.L.); 2 Department of Neurology, Institute of Translational Neurology, Medical Faculty, University Hospital Muenster, 48149 Muenster, Germany

**Keywords:** protein phosphatase 2A (PP2A), influenza A virus (IAV), phosphorylation, apoptosis

## Abstract

Influenza A viruses (IAVs) are respiratory pathogens that are able to hijack multiple cellular mechanisms to drive their replication. Consequently, several viral and cellular proteins undergo posttranslational modifications such as dynamic phosphorylation/dephosphorylation. In eukaryotic cells, dephosphorylation is mainly catalyzed by protein phosphatase 2A (PP2A). While the function of kinases in IAV infection is quite well studied, only little is known about the role of PP2A in IAV replication. Here, we show, by using knockdown and inhibition approaches of the catalytic subunit PP2Ac, that this phosphatase is important for efficient replication of several IAV subtypes. This could neither be attributed to alterations in the antiviral immune response nor to changes in transcription or translation of viral genes. Interestingly, decreased PP2Ac levels resulted in a significantly reduced cell viability after IAV infection. Comprehensive kinase activity profiling identified an enrichment of process networks related to apoptosis and indicated a synergistic action of hyper-activated PI3K/Akt, MAPK/JAK-STAT and NF-kB signaling pathways, collectively resulting in increased cell death. Taken together, while IAV seems to effectively tap leftover PP2A activity to ensure efficient viral replication, reduced PP2Ac levels fail to orchestrate cell survival mechanisms to protect infected cells from early cell death.

## 1. Introduction

Influenza A viruses (IAVs) evoke annually occurring outbreaks of the common flu, which leads to huge economic losses and hundreds of thousands of deaths worldwide. Due to a very limited genome capacity, IAVs hijack host cellular pathways to facilitate their replication and efficiently inhibit antiviral immune responses (reviewed in [1,2,3]). Several studies were able to correlate this ability with dynamic posttranslational modifications (PTMs) of viral as well as cellular targets (reviewed in [4,5,6]). One of the most frequently and intensively studied PTMs is phosphorylation. Different comprehensive studies identified viral as well as cellular proteins to be regulated by phosphorylation in IAV infection [7,8,9]. Most interestingly, in the case of the cellular rapidly accelerated fibrosarcoma/mitogen-activated protein kinase/extracellular signal-regulated kinase (Raf/MEK/ERK) pathway, a biphasic phosphorylation pattern was described in IAV infection [10], indicating that the balance between phosphorylation and dephosphorylation is also crucial for efficient IAV replication. A genome-wide ribonucleic acid interference (RNAi) screen identified cellular cofactors involved in the regulation of kinase and phosphatase activity to be enriched in IAV infected cells [11], which further highlights the importance of phosphorylation and dephosphorylation for IAV infection.

In eukaryotic cells, the activity of several hundreds of specific serine/threonine kinases is mainly counteracted by two phosphatases belonging to the family of phosphoprotein phosphatases: protein phosphatase 1 (PP1) and PP2A [12]. PP2A is the most abundant phosphatase inside eukaryotic cells and was already shown to be activated in primary human monocyte-derived macrophages infected with a highly pathogenic avian influenza virus [13]. PP2A forms heterotrimeric holoenzymes comprised of a catalytic C subunit (PP2Ac) that carries out the dephosphorylation reaction, a scaffolding A subunit building the frame of the complete holoenzyme, and a regulatory B subunit, which is controlling substrate specificity, subcellular localization and the activity of the respective holoenzyme [14]. Thus far, 23 different cellular B subunit isoforms were identified resulting in the formation of approximately 100 different PP2A holoenzymes inside eukaryotic cells [15]. This clearly indicates the involvement of PP2A in a variety of even opposing cellular processes such as the regulation of the activity of different signaling cascades, the control of cell growth and division, or the maintenance of a balance between cell death and survival (reviewed in [16]). PP2A was especially identified to control the activity of several signaling pathways known to be induced in IAV infection such as the Raf/MEK/ERK pathway [10,17] or the phosphoinositide 3-kinases (PI3K)/Akt pathway [18,19]. These functions highlight a possible role of PP2A in IAV infections, which is not completely unraveled thus far.

In this study, we report that the phosphatase activity of PP2A is required for efficient IAV replication. While a reduction in PP2A catalytic activity does not seem to directly modulate viral life cycle progression, PP2A is rather involved in maintaining cell survival of IAV-infected cells.

## 2. Results

### 2.1. PP2Ac Is Needed for Efficient IAV Infection

**Figure 1:**
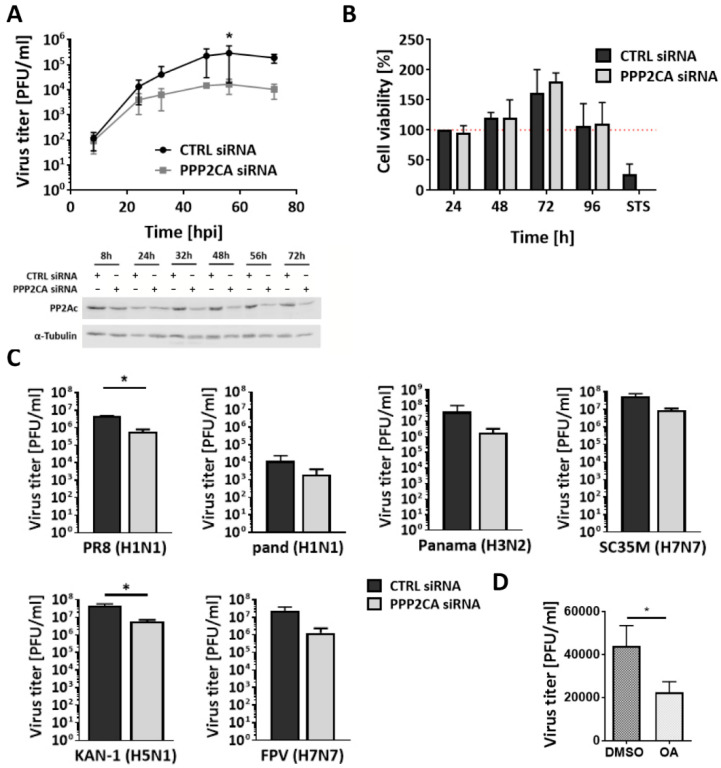
Cellular PP2A is required for efficient IAV replication. (**A**) A549 cells were infected with IAV strain A/WSN/33 (H1N1, WSN) at a multiplicity of infection (MOI) of 0.01, 24 h after siRNA-mediated knockdown (KD) of PP2Ac. Virus-containing supernatants were collected at the indicated time points. The amount of infectious particles was titrated with standard plaque assays. KD efficiency was visualized via Western blotting. (**B**) Cytotoxic effects of the PP2Ac KD were excluded via 3-(4,5-dimethylthiazol-2-yl)-2,5-diphenyltetrazolium bromide (MTT)-based cell viability assay with 1 µM staurosporin (STS) used as positive control. (**C**) A549 cells were infected for 24 h with IAV strains A/Hamburg/04/09 (pand, MOI = 0.1), A/Puerto Rico/8/34 (PR8, MOI = 0.1), A/Panama/2007/99 (Panama, MOI = 0.1), A/SC35M (SC35M, MOI = 0.01), A/Thailand/KAN-1/2004 (KAN-1, MOI = 0.001) and A/FPV/Bratislava/79 (FPV, MOI = 0.001) 48 h after siRNA-mediated KD of PP2Ac. The amount of infectious particles was titrated with standard plaque assays. (**D**) A549 cells were infected with WSN (MOI = 1). Five hpi, 100 nM okadaic acid (OA) or solvent CTRL (DMSO) was added. Supernatants were collected 9 hpi. The amount of infectious particles was titrated with standard plaque assays. (**A**–**D**) Data are represented as means + standard deviation (SD) of three independent experiments with three biological replicates. Statistical significance was analyzed using two-way ANOVA (**A**,**B**) or unpaired two-tailed t-test (**C**,**D**) with * *p* < 0.05.

To investigate the impact of PP2A on IAV replication, we performed an siRNA-mediated knockdown (KD) of the catalytic subunit of PP2A in human lung epithelial A549 cells and subsequently infected these cells with the H1N1 IAV strain A/WSN/33 (WSN) (Figure 1A). A reduction in PP2Ac protein levels led to decreased viral titers by one order of magnitude in comparison to the siRNA CTRL starting at 24 h post infection (hpi). This titer reduction was maintained for the whole course of the experiment with a significant reduction 48 hpi indicating a virus-supportive function of PP2Ac in an IAV infection. KD efficiency was proven using Western blot analysis. Since PP2A is known to be involved in the regulation of several cell cycle-related processes such as cell growth or the balance between cell survival and cell death (reviewed in [16]), cell viability was analyzed after siRNA-mediated KD of PP2Ac to exclude cytotoxic side effects of the KD itself (Figure 1B). Cell viability of both the siRNA PP2Ac- and siRNA CTRL-transfected cells was comparable from 8 to 96 h, indicating that the observed effects on WSN replication are not based on differences in cell viability due to decreased PP2Ac levels. Next, the strain dependency of the observed reduction in virus titer was addressed by infecting siRNA-transfected cells with several low pathogenic human (A/Hamburg/04/09 (pand), A/Puerto Rico/8/34 (PR8), and A/Panama/2007/99 (Panama)) or highly pathogenic avian (A/seal/Mass/1-SC35M/80 (SC35M), A/Thailand/KAN-1/2004 (KAN-1), and A/FPV/Bratislava/79 (FPV)) IAV strains (Figure 1C). Titers of all the virus strains analyzed revealed a tendency of reduced replication in PP2Ac-silenced A549 cells, with PR8 and KAN-1 showing a significantly decreased amount of viral progeny by one order of magnitude. These results clearly indicate that PP2Ac is a crucial factor for the replication of different IAVs. Furthermore, this effect was not specific for A549 cells but was also observed in human bronchial epithelial Calu-3 cells (Supporting information, Figure S1). To validate if the phosphatase activity of PP2A is required for efficient IAV replication, treatment with the well-described PP2A inhibitor okadaic acid (OA, [20]) was used to inhibit all the PP2A holoenzymes present within the cell. Since PP2A is known to play crucial roles during cell cycle regulation, long-term inhibition of PP2A activity can induce cell death. Therefore, treatment conditions were established using an MTT-based cell viability assay (Figure S2). It became apparent that a complete PP2A inhibition by OA for longer than 4 h led to decreased cell viability. As a consequence, the impact of PP2A inhibition on IAV replication was analyzed only within the first replication cycle (9 hpi) with OA being added 5 hpi. Here, a significant reduction in viral titers by 50% was observed (Figure 1D). These results indicate that the PP2A phosphatase activity is indeed essential for efficient IAV replication. Taken together, we conclude that PP2A exhibits a virus-supportive role in IAV infection that depends on its phosphatase activity.

### 2.2. PP2Ac Does Not Alter the Antiviral Immune Response to IAV Infection

**Figure 2:**
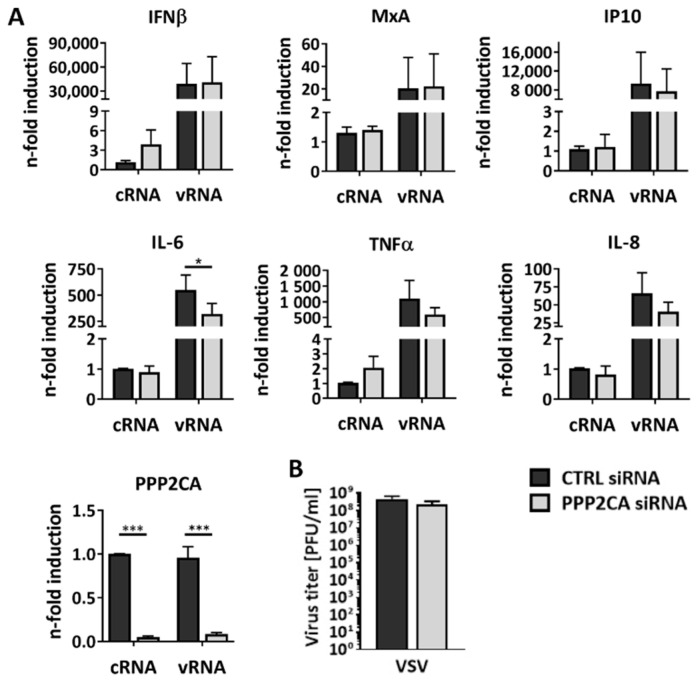
PP2A does not alter the IAV-induced antiviral innate immune response. (**A**) A549 cells were stimulated with previously isolated cellular RNA (cRNA) or viral RNA (vRNA) 48 h after siRNA-mediated KD of PP2Ac. Transcription of exemplary genes of the antiviral IFN-mediated and pro-inflammatory immune response were analyzed using quantitative real-time PCR. KD efficiency was analyzed by measurement of PP2Ac mRNA levels. (**B**) A549 cells were infected with VSV (MOI = 0.01) 48 h after siRNA-mediated KD of PP2Ac. Virus titers were analyzed using standard plaque assays. (**A**,**B**) Data are represented as means + SD of three independent experiments with three biological replicates. Statistical significance was analyzed using a two-way ANOVA (**A**) or unpaired two-tailed t-test (**B**) with * *p* < 0.05 and *** *p* < 0.001.

To unravel the mechanism behind the virus-supportive role of PP2Ac in IAV infection, we first analyzed known PP2A functions related to cellular antiviral responses that are also triggered by IAV infection. Many studies already demonstrated that PP2A regulates the activity of a variety of different cellular kinases involved in mediating antiviral immune responses (reviewed in [16]). To investigate whether PP2A affects the IAV-induced innate antiviral immune response, gene expression levels of several targets such as the cytokine interferon beta (IFNβ) and the subsequently induced IFN-stimulated genes (ISGs) IFN gamma-induced protein 10 (IP10) and antiviral myxovirus resistance protein 1 (MxA) were analyzed in PP2Ac-silenced, WSN-infected cells. However, the expression of antiviral genes was not significantly altered in PP2Ac KD compared to CTRL cells (Figure S3A). Since the observed reduction in virus titers might also affect immune response induction in an infection setting, the effects of PP2Ac KD on the cellular antiviral and pro-inflammatory immune response were analyzed in a limited system. Therefore, only the major pathogen-associated molecular pattern (PAMP), which is viral RNA, was used to stimulate an immune response. Subsequent to PP2Ac silencing, A549 cells were stimulated with previously isolated total RNA from uninfected (cellular RNA, cRNA, as a control) or WSN-infected (viral RNA, vRNA) cells (Figure 2A). As expected, we observed a strong induction of the mRNA levels of all the analyzed cytokines and antiviral genes after vRNA stimulation. However, these levels were not altered in the absence of PP2Ac except for pro-inflammatory cytokine interleukin (IL)-6, which showed a significant two-fold reduction in gene expression. KD efficiency was proven by significantly decreased PP2Ac mRNA levels. In order to further validate that decreased IAV replication in PP2Ac KD cells is independent of alterations in the antiviral immune response, highly IFN-sensitive vesicular stomatitis virus (VSV) was used as a biosensor (Figure 2B). In agreement with our gene expression data, the KD of PP2Ac did not affect VSV replication and, thus, does not seem to interfere with the antiviral IFN-mediated response. To link these results to PP2A activity, we analyzed the expression of antiviral and pro-inflammatory genes after the inhibition of PP2A by OA (Figure S3B). As expected, there was no significant alteration in the induction of these cytokines observable. Taken together, these results clearly show that decreased PP2Ac levels do not affect the innate antiviral immune response of IAV-infected cells but rather mediate decreased IAV replication through other mechanisms.

### 2.3. PP2Ac Does Not Affect Viral Transcription or Translation

**Figure 3:**
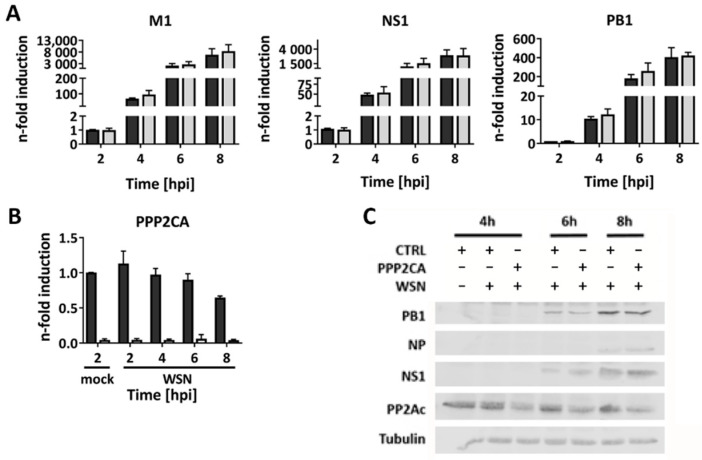
Viral transcription and translation is independent of high PP2Ac levels. (**A**,**B**) A549 cells were infected with WSN (MOI = 5), 48 h after siRNA-mediated KD of PP2Ac. Transcription of exemplary viral mRNAs as well as PP2Ac mRNA as KD control was analyzed using quantitative real-time PCR. Data are depicted as means + SD of three independent experiments with three biological replicates. Statistical significance was analyzed using a two-way ANOVA. (**C**) Expression of exemplary viral proteins was analyzed using Western blotting. Blots are representatives of three independent experiments.

Since we excluded the cellular antiviral immune response to be responsible for decreased IAV replication, we next tried to unravel which step of the viral replication cycle might be regulated by PP2Ac. Therefore, we performed single cycle infections with a high MOI of WSN and analyzed the transcription and translation of exemplary viral genes at the indicated time points (Figure 3). Neither the transcription of the viral mRNAs encoding matrix protein 1 (M1), non-structural protein 1 (NS1) or polymerase basic protein 1 (PB1) (Figure 3A) nor the translation of the viral proteins PB1, nucleoprotein (NP) or NS1 was altered in the PP2Ac-silenced A549 cells in comparison to the CTRL siRNA-transfected cells. Thus, it can be concluded that the early steps of the viral replication cycle are not affected by decreased levels of PP2Ac. To confirm that the transcription and translation of viral proteins do not require PP2A activity, we analyzed the expression of respective viral mRNAs after an OA-mediated inhibition of PP2A 6 hpi (Figure S4). As observed for PP2Ac KD, PP2A inhibition did not alter the expression of viral mRNAs, validating that intermediate steps of viral replication do not require high PP2Ac levels or phosphatase activity.

### 2.4. PP2Ac Does Not Affect Nuclear Export of vRNPs but Rather Regulates Cell Fate in IAV Infection

**Figure 4:**
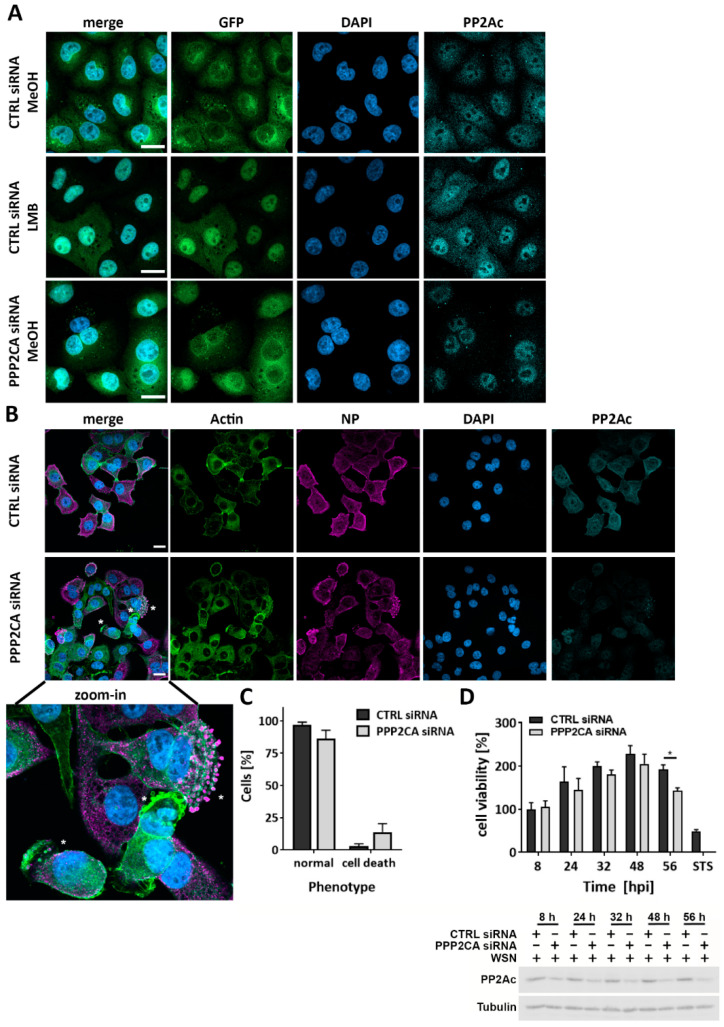
PP2A orchestrates cell survival mechanisms in IAV-infected cells. (**A**) A549 cells stably expressing a GFP biosensor were treated with leptomycin B (LMB) or methanol (MeOH) as solvent control, 48 h after siRNA-mediated KD of PP2Ac. Cells were fixed in 3.7% formaldehyde and nuclei were stained with 4′,6-diamidino-2-phenylindole (DAPI). (**B**,**C**) A549 cells were infected with WSN (MOI = 5) 48 h after siRNA-mediated KD of PP2Ac. Cells were fixed 8 hpi in 3.7% formaldehyde and stained with specific antibodies against the viral nucleoprotein (NP), actin and PP2Ac. Nuclei were stained with DAPI. All immunofluorescence pictures were analyzed with Fiji. Asterisks indicate membrane blebbing. Scale bar corresponds to 20 µm. (**C**) Cells with an obvious cell death phenotype were counted manually and expressed as percent of the total amount of counted cells. Data are represented as mean + SD of three independent experiments with at least 200 analyzed cells each. (**D**) A549 cells were infected with WSN (MOI = 0.01) 24 h after siRNA-mediated KD of PP2Ac. Cell viability was analyzed using an MTT-based assay. Staurosporin (STS, 1 µM) was used as a positive control. KD efficiency was analyzed using Western blotting. Data are depicted as mean + SD of one representative out of four independent experiments. (**C**,**D**) Statistical analysis was performed by using a two-way ANOVA with * *p* < 0.05.

Since we were able to show that neither the cell intrinsic antiviral immune response nor viral transcription and translation were the reason for decreased IAV replication in PP2Ac KD cells, we next presumed that the nuclear export of viral ribonucleoproteins (vRNPs) might be affected, since it is known that this transport process is promoted by the phosphorylation of viral NP [21]. Since vRNPs are known to be exported out of the nucleus in a chromosomal region maintenance 1 (CRM-1)-dependent manner [22], we first analyzed whether PP2Ac KD affects the CRM-1-dependent nuclear export in general. Therefore, we used a stable cell line expressing a translocation biosensor developed by Fetz et al. [23] based on the green fluorescent protein (GFP). GFP was modified to shuttle constantly between nucleus and cytoplasm, whereby the nuclear export is solely mediated by CRM-1. In order to investigate if PP2Ac regulates the CRM-1-dependent nuclear export, we analyzed GFP localization in PP2Ac-silenced cells in comparison to CTRL siRNA-transfected cells (Figure 4A). Interestingly, PP2Ac KD did not lead to GFP accumulation inside the nucleus (lower panel); rather, the GFP biosensor was comparably present inside the nucleus and cytosol as observed in CTRL siRNA, solvent-treated cells (upper panel). This indicates that PP2Ac might also not be involved in the nuclear export of vRNPs. The system was validated by use of leptomycin B (LMB), a well-described specific inhibitor of the CRM-1 pathway, which efficiently retained the GFP biosensor inside the nucleus (middle panel). Interestingly, protein amounts of cytosolic PP2Ac were decreased in PP2Ac KD cells, whereas nucleic proportions of PP2Ac were still present, indicating that our KD setting might still allow for the formation of especially nuclear localized holoenzymes. We confirmed that the CRM-1-mediated nuclear export is not generally affected by the phosphatase activity of PP2A by using OA, which did not alter GFP localization (Figure S5). Next, we analyzed the nuclear export and subsequent transport of vRNPs in the context of genuine IAV infection. It was previously reported that PP2A is an important regulator of the cellular cytoskeleton [24], which, in turn, is known to be exploited by IAVs for efficient transport of viral components required for virus assembly (reviewed in [25]). In order to analyze if the transport of vRNPs might be regulated by PP2A, we investigated the localization of the viral protein NP as an exemplary component of vRNPs at 4, 6 and 8 hpi in siRNA-transfected A549 cells using fluorescence microscopy (Figure S6). While there were no differences in the subcellular localization of NP detectable at the indicated time points, NP aggregates were observed in the membrane region of PP2Ac siRNA-transfected A549 cells at 8 hpi. To examine whether this effect was specific for viral proteins or a general phenomenon, we stained for actin to visualize the cytoskeleton (Figure 4B). In the absence of PP2Ac, not only aggregates of viral NP but also actin were detectable, a phenotype referred to as membrane blebbing (highlighted by asterisks in merge images), which is described to occur during cell death mechanisms such as apoptosis or oncosis [26]. The quantification of membrane blebbing events revealed that in PP2Ac-silenced A549 cells, occurrence of the cell death-like phenotype was enhanced (15%) in comparison to the CTRL siRNA-transfected cells (7%) (Figure 4C). In order to investigate whether this phenotype can be linked to cell death, we analyzed changes in the cell viability of IAV-infected, PP2Ac siRNA-transfected cells (Figure 4D). Starting from 24 hpi, the same time point where a reduction in virus titers was first visible, we observed an up to 30% decreased cell viability in the infected PP2Ac-silenced cells compared to the CTRL siRNA-transfected cells.

Thus, we conclude that the observed effects on IAV replication result from a dysregulation of the cell survival/cell death balance due to decreased PP2Ac levels.

### 2.5. PP2Ac Is Involved in Cell Survival Mechanisms by Decreasing the Activation of Different Signaling Hubs after IAV Infection

**Figure 5:**
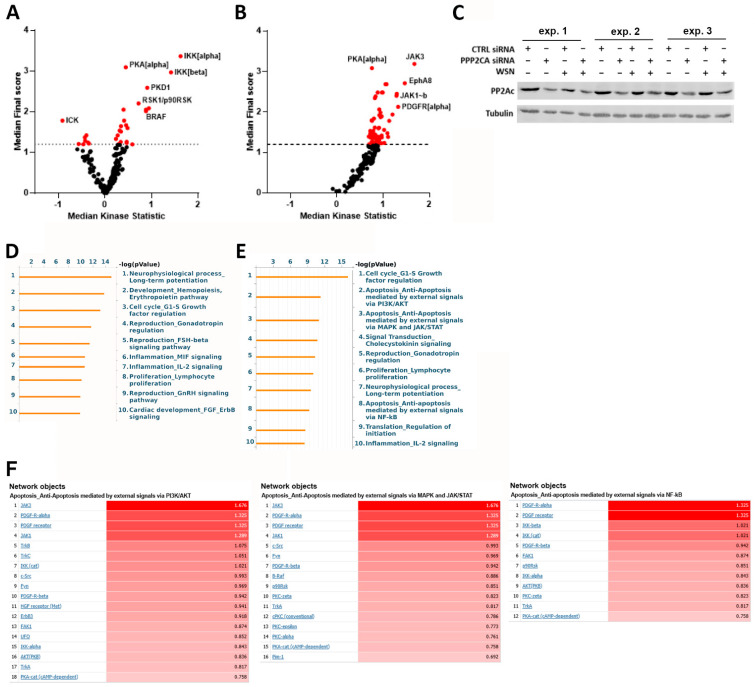
PP2A controls different signaling pathways decisive for cell survival in IAV infection. (**A**–**E**) A549 cells were mock-infected (**A**) or infected with WSN (MOI = 5) (**B**) 48 h after PP2Ac KD, and kinase activity was assessed 8 hpi by chip-based kinase activity profiling (PamGene technology). Differences in kinase activity in PP2Ac-silenced compared to CTRL siRNA-transfected cells are depicted as median kinase statistic (negative values = lower activity, positive values = higher activity) of three independent experiments. (**C**) Efficient PP2Ac KD in independent experiments was confirmed using Western blotting. (**D**,**E**) Network process enrichment analyses of differentially activated kinases obtained in mock-infected (**D**) or IAV-infected (**E**) cells were computed using MetaCore. (**F**) Lists of network objects of apoptotic processes and their differential activation.

Since PP2A seems to regulate the intimate balance between cell survival and cell death after IAV infection, we wanted to obtain a comprehensive insight into the specific pathways modified by PP2A that are decisive for cell fate in IAV infection. The activation landscape of various kinases was screened by using a fluorescence-based kinase activity profiling assay applied 8 hpi. In order to obtain detailed information about the role of PP2A within uninfected and IAV-infected cells, we compared the kinase activity profiles of CTRL siRNA- with PP2Ac siRNA-transfected cells, respectively (Figure 5A,B). Efficient PP2Ac KD in all three experiments was confirmed using Western blot (Figure 5C) and variation between biological samples was evaluated using coefficient of variation (CV) plots (Figure S7). In uninfected cells, some kinases such as intestinal cell kinase (ICK) were less active (negative median kinase statistic) with decreased levels of PP2Ac, whereas a larger number of kinases such as IκB kinase (IKK)α and IKKβ showed increased activity in these cells (positive median kinase statistic). Interestingly, in IAV-infected cells, the activity of nearly all screened kinases was increased in PP2Ac KD cells, indicating an important role for PP2A in the control of IAV-induced signaling pathways. To narrow down which biological processes might be affected most, we applied an integrated network analysis by using the web-based database MetaCore. As expected, within the top three network processes identified based on differentially activated kinases in PP2Ac KD cells, cell cycle control and especially G1-S growth factor regulation was enriched in uninfected (Figure 5D) as well as IAV-infected cells (Figure 5E). Of note, three of the enriched network processes in IAV infection were directly linked to apoptotic cell death, which correlates to our previous observation that PP2Ac KD decreases cell viability after IAV infection. Here, apoptosis regulation seems to be mainly regulated by PI3K/Akt, MAPK (mitogen-activated protein kinase) and janus kinase (JAK)/STAT (signal transducers and activators of transcription) as well as NF-ĸB (nuclear factor kappa-light-chain-enhancer of activated B-cells) signaling pathways. A closer look into the network objects present in these apoptotic processes revealed a major role for hyper-activated platelet-derived growth factor receptor (PDGFR), JAK3 and JAK1, IKKs as well as Akt (Figure 5F), collectively orchestrating apoptosis control in PP2Ac KD cells after IAV infection.

### 2.6. PP2Ac Is Involved in Apoptotic Cell Death Control in IAV Infection

**Figure 6:**
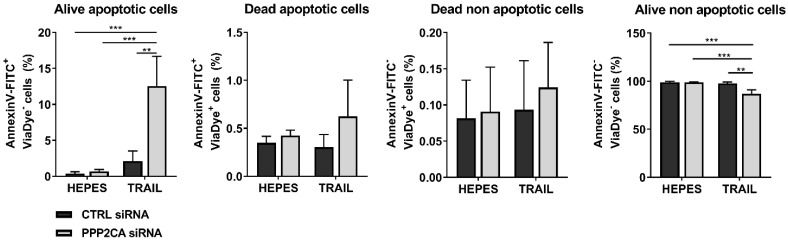
PP2A confines apoptotic cell death. PP2Ac siRNA- or CTRL siRNA-transfected A549 cells were treated with 50 ng/mL TRAIL diluted in HEPES (4-(2-hydroxyethyl)-1-piperazineethanesulfonic acid). The amount of apoptotic cells was analyzed 4.5 h post stimulation by a combinatory staining of Viability Dye (life/dead marker) and Annexin V by using flow cytometry. Data are represented as means + SD of three independent experiments with three biological replicates. Statistical significance was analyzed using a two-way ANOVA with ** p < 0.01 and *** *p* < 0.001.

Since the comprehensive kinase activity profiling clearly indicated a hyper-activation of kinases involved in apoptotic cell death mechanisms, we analyzed PP2A-mediated apoptosis control after stimulation with TRAIL (tumor necrosis factor related apoptosis-inducing ligand). It was described previously that IAV infection leads to an induction of death receptor-mediated signaling via TRAIL induction [27]. Here, we applied a combinatory staining of Annexin V, which binds to externalized phosphatidylserine in apoptotic cells, and Fixable Viability Dye, which solely stains dead cells and, thus, allows for the discrimination of living and dead cells (Figure 6). TRAIL stimulation led to a significantly elevated apoptosis induction in PP2Ac KD cells (12%) in comparison to 3% apoptotic cells in CTRL siRNA-transfected cells, indicating that PP2A is involved in the orchestration of cell survival mechanisms and the confinement of apoptotic signaling pathways in scenarios of apoptosis induction, such as IAV infections.

## 3. Discussion

IAVs are still a major threat to the human population and are responsible for many deaths worldwide. To efficiently target IAVs and thus improve the course of disease, it is important to understand the complex mechanisms of interaction between IAVs and the respective host cells. In this study, we identified cellular phosphatase PP2A to play an important virus-supportive role by mainly interfering with the balance of cell survival/cell death following an IAV infection. The reduced levels of PP2Ac led to a decreased cell viability of the IAV-infected cells, supposably mediated by a hyper-activation of apoptosis regulating pathways PI3K/Akt, MAPK and JAK/STAT as well as NF-ĸB, ultimately resulting in apoptosis induction and an interference with efficient IAV replication.

PP2A is an important regulator of several cellular mechanisms, such as the cell cycle and cell growth, or the regulation of signaling pathways involved in other cellular processes. It is not surprising that PP2A was also identified as a direct or indirect factor involved in viral infections. Several viruses exploit cellular PP2A to actively influence their replication. In the case of Ebola virus infection, the transcription of the viral genome depends on the dephosphorylation of the viral transcription factor VP30, which is catalyzed by recruited PP2A, mediated by Ebola virus protein NP [28]. The small T-antigen of polyomaviruses directly inhibits PP2A by replacing the regulatory subunit [29,30], which subsequently enhances viral replication [31]. For the human respiratory syncytial virus, it was shown that PP2A is involved in the regulation of the phosphorylation status of the viral P-protein [32]. Along that line, PP2A is also involved in the efficient replication of IAVs, as was shown in this study. However, in contrast to our expectations, reduced PP2A levels do not seem to affect IAV transcription or translation. However, this observation might be explained by the fact that leftover PP2A seems to accumulate in the nucleus of PP2Ac-silenced cells where IAV genome transcription and replication takes place. Alternatively, other phosphatases might be involved in these steps such as, for example, PP6, which was shown to directly interact with and positively regulate the IAV polymerase [33].

In addition, in the context of IAV infection, IFNβ expression and induction of the IFN-mediated antiviral immune response were not altered in PP2Ac KD cells. This result was rather surprising since, in previous studies, it was shown that PP2A is involved in the regulation of antiviral responses. In the case of hepatitis C virus infection, PP2A impairs the virus-induced antiviral IFNα-mediated immune response, which was speculated to ultimately facilitate the establishment of a chronic infection [34]. Furthermore, PP2Ac deficiency in macrophages was associated with increased type I IFN signaling after poly(I:C) treatment [35], a commonly used double-stranded RNA analog to simulate virus infections. The fact that PP2Ac KD has a different impact on type I IFN signaling in human lung epithelial cells and primary macrophages might result from the strength of the antiviral response induced by an IAV infection. A previous study demonstrated that alveolar macrophages challenged with IAV infection almost exclusively activate the IFN-dependent antiviral response, whereas airway epithelial cells induce a much broader IFN-dependent and IFN-independent antiviral response [36]. In addition, the influence of different PP2A holoenzymes on the regulation of these cellular pathways might differ between cell types as well as primary cells and cell lines. A detailed analysis of the human proteome assessable via the Human Protein Atlas (http://www.proteinatlas.org, access date: 25 April 2021) showed that exemplary subunits of PP2A are expressed in all tissues and cell lines, but can vary in their expression level [37,38]. Consequently, there might be a cell line-specific PP2A holoenzyme landscape resulting in a discrete PP2A activity and regulation of particular cellular pathways, contributing to distinct antiviral immune responses in airways epithelial cells versus macrophages.

Ultimately, we hypothesize that the PP2Ac KD-mediated decrease in IAV replication can be linked to a dysregulation of the cell survival/cell death balance. Maintenance of this balance is highly regulated via a network of complex survival, pro- and anti-apoptotic pathways, which are activated in a stimulus dependent manner. The complexity is highlighted by the diverse roles PP2A plays in this network. On the one hand, many studies linked the inhibition of PP2A to the malignant cell growth of cancer cells, and, in turn, could demonstrate that activation of PP2A leads to apoptotic cell death (reviewed in [39]). On the other hand, PP2A inhibition by the compound LB-100 sensitized triple-negative breast cancer cells for treatment with chemotherapeutic drugs by induction of mitotic catastrophe followed by cell death [40]. In this context, the kinases Akt and PLK1 (polo-like-kinase 1) were phosphorylated enabling the induction of mitotic catastrophe. Another study demonstrated that PP2A inhibition by LB-100 sensitized hepatocellular carcinoma cells for treatment with the licensed anti-cancer drug sorafenib by inducing a hyper-phosphorylation of SMAD3 (mothers against decapentaplegic homolog 3), which, in turn, leads to the downregulation of B-cell lymphoma 2 (Bcl-2) resulting in the induction of apoptosis [41]. Taking into account the results of the kinase activity profiling showing that the activity of members of the PI3K/Akt signaling pathway were enriched in PP2Ac KD cells after IAV infection, it seems likely that PP2A KD or inhibition might also sensitize lung epithelial cells to induce cell death after IAV infection. In general, the balance between cell survival and cell death involves an intimate interplay of a complex network of several pro- and anti-apoptotic factors, whose activity is regulated by a huge variety of different mechanisms including de-/phosphorylation in dependence of the type of stimulus [42]. The complexity of these mechanisms is highlighted by the fact that depending on the phosphorylation site and localization, the dephosphorylation of, for example, Bcl-2, which is catalyzed by PP2A, can lead to pro- or anti-apoptotic signaling [43,44]. PP2A or often even specific PP2A holoenzymes were already linked to different cell death mechanisms including their role in mediating the activity of anti- and pro-apoptotic members of the Bcl-2 family (reviewed in [45]).

Previously, it was demonstrated that viruses can specifically target PP2A to negatively regulate cell survival and enable cell death in late stages of the viral life cycle to efficiently release progeny viruses from infected cells. For adenoviruses, it was shown that they manipulate PP2A to induce a p53-dependent apoptotic pathway [46]. Additionally, IAVs are known to manipulate cell death mechanisms. In the initial stages of viral replication, early cell death is prevented by the activity of the anti-apoptotic PI3K/Akt signaling axis, whereas in later stages, apoptosis is induced in a p53- and NF-kB-dependent manner [27,47,48]. Furthermore, MAPKs JNK and p38 have already been linked to apoptosis induction in IAV infection (reviewed in [49,50,51], [52,53]). Interestingly, IAV infection was shown to simultaneously induce autophagy and apoptosis in A549 cells, with autophagy having a regulatory role in virus-induced apoptosis [54]. Signaling pathways known to control autophagy in IAV infection include the PI3K/Akt signaling pathway as well as MAPK JNK [55]. PP2A was already shown to mediate apoptosis and autophagic cell death in multiple myeloma cell lines [56] and, thus, might also affect the crosstalk between these cellular processes in IAV infection. The tight interplay between different signaling cascades activated in IAV infections is of major importance for efficient viral replication [57]. The hyper-activation of these pathways due to limited control by PP2A-mediated dephosphorylation seems to be highly feasible to result in changes in cell fate. Thus, alterations in the cellular phosphorylation landscape induced by reduced PP2A phosphatase activity seem to disrupt the signaling required by the virus to drive efficient replication.

Taken together, PP2A is involved in the regulation of a variety of different cellular mechanisms and signaling pathways required for cell survival. In this study, we were able to demonstrate that cellular PP2Ac is also needed in IAV infection. Here, PP2A plays a virus-supportive role by orchestrating cell survival mechanisms and preventing the hyper-activation of apoptosis-inducing pathways resulting in the protection of infected cells from early cell death, thus enabling efficient IAV replication.

## 4. Materials and Methods

### 4.1. Cell Lines, Virus Strains and Plasmids

Human alveolar epithelial A549 cells and Madin-Darby canine kidney (MDCKII) cells were originally purchased from ATCC and cultivated in Dulbecco’s Modified Eagle’s Medium (DMEM, Sigma-Aldrich, Taufkirchen, Germany) supplemented with 10% (*v*/*v*) FCS (Biochrom, Berlin, Germany) or Minimum Essential Medium Eagle (MEM, Sigma-Aldrich, Taufkirchen, Germany) supplemented with 5% (*v*/*v*) FCS, respectively.

IAV H1N1 strain A/WSN/33 was taken from the virus collection of the Institute of Virology Muenster. Vesicular stomatitis virus strain Indiana (VSV) was kindly provided by Thorsten Wolff (Robert-Koch Institute, Berlin, Germany). A/Thailand/KAN-1/2004 (KAN-1) was used with kind permission from P. Puthavathana (Bangkok, Thailand). A/FPV/Bratislava/79 (FPV) was acquired from the Institute of Virology in Giessen, Germany. Recombinant A/seal/Mass/1-SC35M/80 (SC35M), A/Hamburg/04/09 (pand), A/Panama/2007/99 (Panama) and A/Puerto Rico/8/34 (PR8) were generated using the pHW2000 reverse genetics system [58]. All viruses were propagated on MDCKII cells.

The translocation biosensor construct (20) was kindly provided by Roland Stauber (University of Mainz, Mainz, Germany). Stable cell lines were generated by retroviral transduction of A549 cells. Retroviruses were generated by transfecting HEK293T-Phoenix packaging cells with the retroviral construct. Supernatants were harvested after 72 and 96 h and supplemented with 4 µg/mL polybrene (Santa Cruz Biotechnology, Dallas, TX, USA) and afterwards they were used to transduce A549 cells. GFP-expressing cells were sorted using a SH800 Cell Sorter (Sony, Weybridge, UK).

### 4.2. Virus Infection and Plaque Titration

The infection of A549 cells and plaque titration using standard plaque assays in MDCKII cells were performed as described previously for HEK293 cells [59].

### 4.3. Small Interfering RNA (siRNA)-Mediated KD and RNA Stimulation

To silence PP2Ac expression and to stimulate A549 cells with previously isolated RNA, the transfection reagent Lipofectamine^®^ 2000 (Invitrogen, Carlsbad, CA, USA) was used according to the manufacturer’s instruction. For silencing PP2Ac by an siRNA-mediated KD, A549 cells were transfected in 6 wells with 5 pmol per 3 × 10^5^ cells of PPP2CA-targeting siRNA (PPP2CA siRNA, 5′GAACUUGAGGAUACUCUAA3′) or scrambled siRNA (CTRL siRNA, 5′UUCUCCGAACGUGUCACGU3′) in Opti-MEM (Gibco, Thermo Fisher Scientific, Waltham, MA, USA). For transfection in 12 wells or 24 wells, the amounts for 6 wells were divided by 2 or 3, respectively. The medium was exchanged to DMEM containing 0.2 % (*w*/*v*) bovine serum albumin (BSA, Sigma-Aldrich, Taufkirchen, Germany), 1 mM MgCl_2_ (Carl Roth, Karlsruhe, Germany), 0.9 mM CaCl_2_ (Carl Roth, Karlsruhe, Germany), 100 U/mL penicillin (Biochrom, Berlin, Germany), 0.1 mg/mL streptomycin (Biochrom, Berlin, Germany) 24 h post transfection. To silence PP2Ac expression in Calu-3 cells, 3 × 10^5^ Calu-3 cells were seeded into 12-well plates and incubated for 48 h. Cells were transfected with 50 pmol PPP2CA-targeting siRNA or scrambled siRNA in OptiMEM (Gibco, Thermo Fisher Scientific, Waltham, MA, USA) using Lipofectamine^®^ RNAiMAX (Invitrogen, Carlsbad, CA, USA) according to the manufacturer’s instructions. After an incubation for 24 h, cells were transfected a second time with the same conditions.

To stimulate cells with viral RNA (vRNA) or cellular RNA (cRNA), total RNA was isolated from uninfected (cRNA) or virus-infected (vRNA) cells using peqGOLD TriFast™ (Peqlab Biotechnology GmbH, Erlangen, Germany) according to the manufacturer’s instruction. Stimulation was performed with 200 ng c/vRNA per 3 × 10^5^ A549 cells and lysates were taken 4 h post transfection to use for quantitative real-time PCR (qRT-PCR).

### 4.4. Cell Treatment

A549 cells were treated with different inhibitors according to the experimental design.

PP2A inhibition in A549 cells was achieved by treatment with 100 nM OA (LC Laboratories, Woburn, MA, USA), a well described PP2A inhibitor, for no longer than 4 h. As a control, the same volume of the solvent DMSO (Carl Roth, Karlsruhe, Germany) was used.

Inhibition of the CRM-1-dependent nuclear export was induced by treatment with 5.55 ng/mL LMB (Sigma-Aldrich, Taufkirchen, Germany) for 3 h and the same volume of MeOH (Roth) as corresponding solvent CTRL.

Induction of cell death was initiated by use of 50 ng/mL recombinant human SuperKillerTRAIL™ (Enzo Life Sciences, Lörrach, Germany) versus the same volume of the solvent KillerTRAIL™ Storage and Dilution Buffer (Enzo Life Sciences, Lörrach, Germany). Cells were treated for 4.5 h prior to fluorescence-activated cell sorting (FACS)-based analysis.

### 4.5. Cell Viability Assay

To analyze cell viability, a 3-(4,5-dimethylthiazol-2-yl)-2,5-diphenyltetrazolium bromide (MTT, Sigma-Aldrich, Taufkirchen, Germany)-based assay was used. At indicated time points, 5 mg/mL MTT was added to the supernatant and the cells were incubated for an additional 12 h at 37 °C and 5% CO_2_. Afterwards, the supernatant was aspirated and cells were lysed in DMSO. Subsequently the OD_562 nm_ was measured in technical duplicates with a SpectraMax M2 Microplate reader (Molecular Devices, Sunnyvale, CA, USA). As positive cell death-inducing control 1 µM staurosporin (Sigma-Aldrich, Taufkirchen, Germany) was used.

### 4.6. Total RNA Isolation, Reverse Transcription and qRT-PCR

At indicated time points, total RNA was extracted with the Monarch total RNA Miniprep Kit (New England Biolabs GmbH, Frankfurt am Main, Germany) according to the manufacturer’s instruction for in-well lysis of adherent cells. RNA concentration was measured with a NanoDrop NP-1000 (Peqlab Biotechnology GmbH, Erlangen, Germany) and 1 µg of total RNA was used for reverse transcription. Therefore, the Revert Aid H minus reverse transcriptase (Thermo Fisher Scientific, Waltham, MA, USA) was used according to the manufacturer’s instruction with 0.5 µg of Oligo(dT) primers. The cDNA was further diluted 1:14.5 in ddH_2_O and used for qRT-PCR with the reagent Brilliant III Ultra-Fast SYBR QPCR (Agilent Technologies, Ratingen, Germany) according to the manufacturer’s instruction. For detection of several genes, specific primers against the cellular targets glyceraldehyde 3-phosphate dehydrogenase (GAPDH, fwd 5′GCAAATTCCATGGCACCGT3′; rev 5′GCCCCACTTGATTTTGGAGG3′), IFNβ (fwd 5′GAGAAG5′GAGAAGCACAACAGGAGAGCAA3′; rev 5′TCTGGCACAACAGGTAGTAGGC3′), MxA (fwd 5′GAAGGGCAACTCCTGACAGT3′; rev 5′GTTTCCGAAGTGGACATCGCA3′), IP10 (fwd 5′GGAACCTCCAGTCTCAGCACCA3′; rev 5′AGACATCTCTTCTCACCCTTC3′), IL-6 (fwd 5′AGAGGCACTGGCAGAAAACAAC3′; rev 5′AGGCAAGTCTCCTCATTGAATCC3′), IL-8 (fwd 5′ACTGAGAGTGATTGAGAGTGGAC3′; rev 5′AACCCTCTGCACCCAGTTTTC3′), TNFα (fwd 5′ATGAGCACTGAAAGCATGATC3′; rev 5′GAGGGCTGATTAGAGAGAGGT3′) and PPP2CA (fwd 5′AGCCCCATGTTGTTCTTTGTT3′; rev 5′AGTGCCCTTGATTTTTATTTTACC3′), and the viral targets M1 (fwd 5′AAATGGCTGGATCGAGTGAG3′; rev 5′GCCTGGCCTGACTAGCAATA3′), NS1 (fwd 5′GAGGACTTGAATGGAATGATAACA3′; rev 5′GTCTCAATTCTTCAATCAATCAACCATC3′) and PB1 (fwd 5′CATACAGAAGACCAGTCGGGAT3′; rev 5′GTCTGAGCTCTTCAATGGTGGA3′) were used. Threshold cycle (CT) values were obtained using a Light Cycler 480 (Roche, Mannheim, Germany) with Light Cycler^®^480 Software. All values were normalized to mRNA levels of GAPDH and quantified using the 2^−ΔΔCT^ method [60].

### 4.7. Western Blot Analysis

To analyze protein expression, cells were lysed at indicated time points in radio-immunoprecipitation assay lysis buffer (25 mM Tris-HCl (Carl Roth, Karlsruhe, Germany), 137 mM NaCl (Carl Roth, Karlsruhe, Germany), 10% (*v*/*v*) glycerol (Carl Roth, Karlsruhe, Germany), 0.1% (*w*/*v*) SDS (Carl Roth, Karlsruhe, Germany), 0.5% (*w*/*v*) Deoxycholic acid (Carl Roth, Karlsruhe, Germany), 1% (*v*/*v*) NP-40 (Carl Roth, Karlsruhe, Germany), 2 mM EDTA (Carl Roth, Karlsruhe, Germany), pH 8.0) freshly supplemented with 5 mg/mL leupeptin (Sigma-Aldrich, Taufkirchen, Germany), 5 mg/mL aprotinin (Carl Roth, Karlsruhe, Germany), 0.2 mM Pefabloc (Sigma-Aldrich, Taufkirchen, Germany), 1 mM sodium vanadate (Sigma-Aldrich, Taufkirchen, Germany) and 5 mM benzamidine (Sigma-Aldrich, Taufkirchen, Germany). Cell debris were removed by a 15-min centrifugation at 14,000 rpm and 4 °C. The protein concentration was adjusted equally using a Bradford Assay (Protein Assay Dye Reagent Concentrate (Bio-Rad Laboratories, Feldkirchen, Germany)). Proteins were denaturized in 5 × Laemmli buffer (250 mM Tris-HCl, 8% (*w*/*v*) SDS, 40% (*v*/*v)* glycerol, 10% (*v*/*v*) β-Mercaptoethanol (Carl Roth, Karlsruhe, Germany), 0.01% (*w*/*v*) bromophenol blue (Carl Roth, Karlsruhe, Germany), pH 6.8) for 5 min at 95 °C and subsequently separated on polyacrylamid gels. Afterwards, proteins were electroblotted onto a nitrocellulose membrane using wet transfer. Membranes were blocked in 5% BSA diluted in TBS-T buffer (50 mM Tris-HCl, 150 mM NaCl, 0.2% Triton X-100 (Carl Roth, Karlsruhe, Germany), pH 7.5) and specific protein bands were detected with primary antibodies against PP2Ac (clone 1D6, 1:1000, Merck, Darmstadt, Germany), α-Tubulin (clone DM1A, 1:1000, Sigma-Aldrich, Taufkirchen, Germany), PB1 (GTX125923, 1:1000 GeneTex, Irvine, CA, USA), NP (GTX125989, 1:1000, GeneTex, Irvine, CA, USA) and NS1 (GTX125990, 1:1000, GeneTex, Irvine, CA, USA) and secondary antibodies anti-mouse or anti-rabbit IgG conjugated to the fluorophores 800CW or 680RD (Li-Cor Biosciences, Lincoln, NE, USA, 1:2000). Images were taken with the imaging system Odyssey Fc (Li-Cor Biosciences, Lincoln, NE, USA).

### 4.8. Immunofluorescence (IF) Staining

For IF staining, 5 × 10^4^ A549 cells were seeded and infected on cover slips in 24 wells. At indicated time points, cells were washed with PBS and fixed for 20 min in 3.7% (*v*/*v*) formaldehyde (Carl Roth, Karlsruhe, Germany, diluted in PBS). After two washing steps with PBS, the cells were permeabilized for 5 min with freshly prepared 0.3% (*v*/*v*) Triton X-100 and, after additional washing, blocked with 5% (*w*/*v*) BSA (in PBS). After blocking, internal proteins were stained for 1 h with specific primary antibodies against NP (GTX125989, 1:1000, GeneTex, Irvine, CA, USA) and PP2Ac (clone 1D6, 1:100, Merck, Darmstadt, Germany) and for additional 30 min with the secondary antibodies’ goat anti-mouse IgG (H+L) Alexa 647 (Thermo Fisher Scientific, Waltham, MA, USA, 1:2000), and goat anti-rabbit IgG (H+L) Cross-Adsorbed Secondary Antibody, Alexa Fluor 568 (Thermo Fisher Scientific, Waltham, MA, USA, 1:2000) in the dark. Actin was stained 30 min in the dark with CF^®^488A Phalloidin (biotum, 1:40) and nuclei were visualized with 4′,6-diamidino-2-phenylindole (DAPI, Thermo Fisher Scientific, Waltham, MA, USA, 1:10,000). Cover slips were mounted on microscopy slides with Fluorescent Mounting Medium (Dako, Agilent Technologies, Ratingen, Germany) and analyzed with a laser scanning microscope (LSM 780, Zeiss, Oberkochen, Germany) equipped with a Plan-Apochromat 63x (NA 1.4) oil immersion objective (Zeiss, Oberkochen, Germany). All images were analyzed with the program Fiji (ImageJ).

### 4.9. FACS Staining

For FACS-based analysis of cell death mechanisms, 1.5 × 10^5^ A549 cells were transfected per 12-well. Supernatants of TRAIL-treated A549 cells were collected and recombined with the respective detached cells. These cells were washed with serum-free PBS and stained for 30 min in the dark with FITC-labelled Annexin V (31490013 × 2, 1:20, Immunotools, Friesoythe, Germany) and ebioscience Fixable Viability Dye eFluor 450 (1:2000, Invitrogen, Carlsbad, California, USA). Afterwards, cells were fixed for 10 min with 4% (*w*/*v*) paraformaldehyde (Sigma-Aldrich, Taufkirchen, Germany) diluted in PBS and then resuspended in 250 µL of annexin V staining buffer (0.01 M HEPES, 0.14 M NaCl and 2.5 mM CaCl2, pH 7.5). Flow cytometric measurements were performed with a Gallios flow cytometer (Beckman Coulter, Indianapolis, Indiana, USA). Data were analyzed using the FlowJo software (v.10, BD Biosciences, Mississauga, ON, USA).

### 4.10. Kinase Activity Profiling

A549 cells were transfected with PPP2CA-targeting or scrambled siRNAs, respectively, as described previously. Forty-eight h post transfection, cells were infected with WSN at a MOI of 5 and lysed 8 hpi. Cells were washed twice with ice cold PBS and lysed with ice cold M-PER^TM^ Mammalian Extraction Buffer (Thermo Fisher Scientific, Waltham, MA, USA) supplemented with Halt^TM^ Phosphatase Inhibitor Cocktail (100×) and EDTA free Halt^TM^ Protease Inhibitor Cocktail (100×) (Thermo Fisher Scientific, Waltham, MA, USA). Lysates were centrifuged at 10,000 rpm for 15 min at 4 °C and cleared supernatants were aliquoted and frozen at −80 °C. Protein concentrations were measured by using Bradford reagent (Bio-Rad Laboratories, Feldkirchen, Germany) following the manufacturer’s instructions. For the protein tyrosine kinase (PTK) array protocol (v04), 5 μg of protein extract was used, and 1 μg for the serine/threonine kinase (STK) array protocol (v11). Measurements were performed on a PamStation^®^12 from PamGene (‘s-Hertogenbosch, The Netherlands). Briefly, the PTK array was processed in a single-step reaction. Cell extracts, ATP and FITC-labelled pY20 antibodies were incubated on the chip and the phosphorylation of the individual Tyr-peptides was followed by fluorescence detection in real time. The STK array was processed in a two-step reaction. First, cell lysates, ATP and the primary antibody mixture was incubated on the chip for 110 min. Second, the reaction mix was removed and secondary FITC-labelled antibodies were added. Development of the fluorescence signal was detected using Alexa488 fluorescence. Signal intensities and correlation to kinase activity were analyzed using BioNavigator6 v06.03.63.0 (PamGene, ‘s-Hertogenbosch, The Netherlands). Process networks of kinases with high sensitivity (median final score > 1.2) were computed using MetaCore, a Cortellis Solution by Clarivate Analytics (London, UK).

## Data Availability

All data presented in this study are available in the article and its Supplementary Materials.

## Supplementary Materials

The following are available online at https://www.mdpi.com/article/10.3390/ijms222011164/s1, Figure S1: Cellular PP2A is required for efficient IAV replication in Calu-3 cells, Figure S2: Cytotoxic effects of varying concentrations of the inhibitor okadaic acid (OA), Figure S3: PP2A does not alter the IAV- induced innate immune response, Figure S4: Inhibition of PP2A activity does not alter the transcription of viral genes, Figure S5: PP2A does not affect the CRM-1-dependent nuclear export, Figure S6: PP2A does not affect trafficking of viral RNP complexes but induces an increased cell death phenotype in IAV-infected cells, Figure S7: Biological samples used for kinase activity profiling show low variance.
