# Supplementary material for: Cellular Protein Phosphatase 2A Regulates Cell Survival Mechanisms in Influenza A Virus Infection
Source: Int J Mol Sci. 2021 Oct 16;22(20):11164. doi: 10.3390/ijms222011164 (PMC8540457; doi:10.3390/ijms222011164)
Supplement: Supplementary file 1 [file ijms-22-11164-s001.zip › ijms-1402924-supplementary/Supplementary Information.pdf]

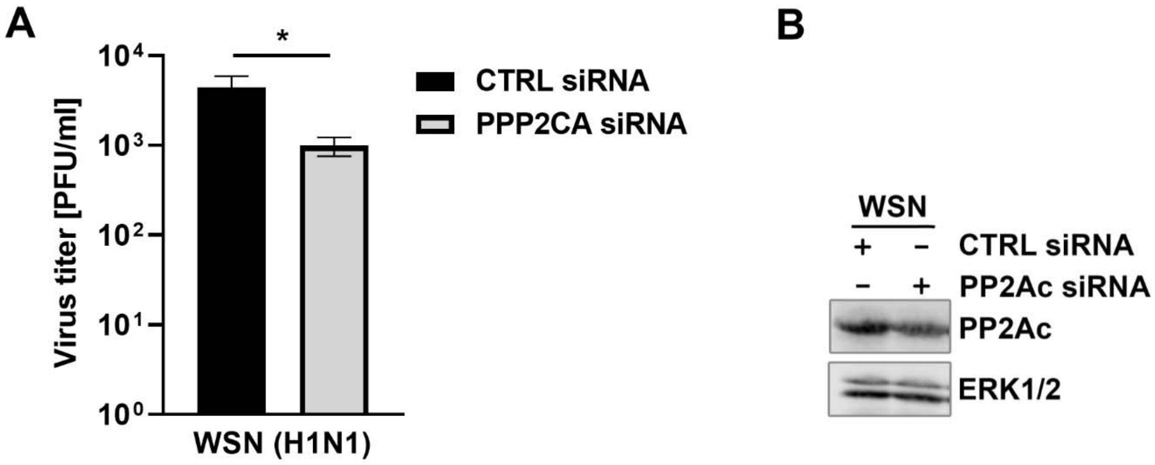

Figure S1: Cellular PP2A is required for efficient IAV replication in Calu-3 cells. A) Calu-3 cells were infected with IAV strain A/WSN/33 (H1N1, WSN) at a multiplicity of infection (MOI) of 0.01, 24 h after siRNA-mediated knockdown (KD) of PP2Ac. Amount of infectious particles was titrated with standard plaque assays. Data are depicted as means + standard deviation (SD) of one representative of two independent experiments. Statistical significance was analyzed by unpaired two-tailed t-test with * $p < 0.05$. B) PP2Ac KD efficiency was visualized via western blotting.

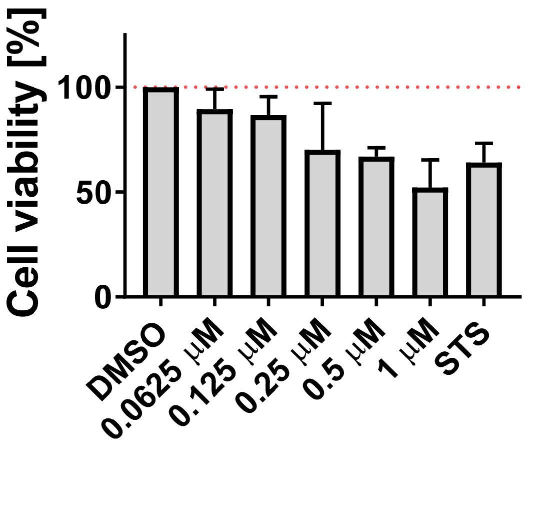

Figure S2: Cytotoxic effects of varying concentrations of the inhibitor okadaic acid (OA). A549 cells were treated with the indicated concentrations of OA. Cell viability was analyzed 6 h post stimulation via MTT-based cell viability assay. Treatment with 1 µM staurosporin (STS) was used as positive control. Data are presented as means + SD of three independent experiments.

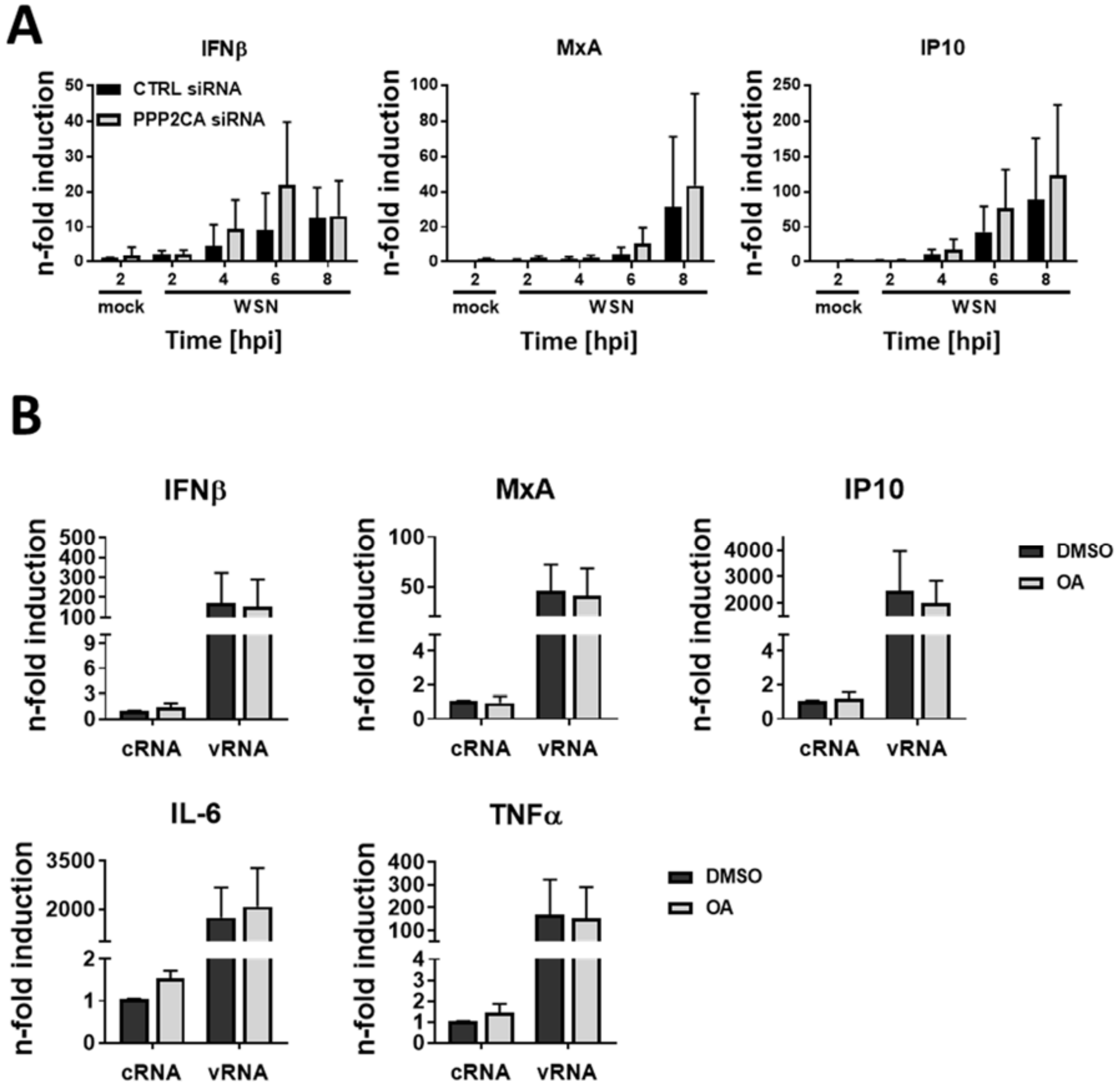

Figure S3: PP2A does not alter the IAV- induced innate immune response. A) A549 cells were infected with A/WSN/33 (H1N1, WSN, MOI = 5) 48 h after siRNA-mediated knockdown (KD) of PP2Ac. B) A549 cells were stimulated for 3 h with 200 ng of previously isolated cellular RNA (cRNA) or viral RNA (vRNA) in the presence of 100 nM okadaic acid (OA) or DMSO as solvent control. A, B) Transcription of exemplary genes of the antiviral interferon (IFN)-mediated immune response were analysed by quantitative real-time PCR. Data are presented as means + SD of three independent experiments with three biological replicates. Statistical significance was analyzed by two-way ANOVA.

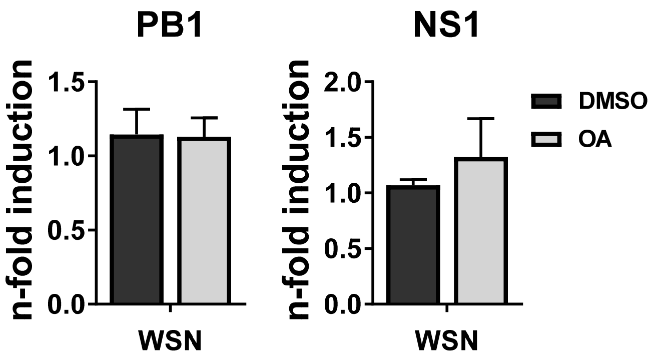

Figure S4: Inhibition of PP2A activity does not alter the transcription of viral genes. A549 cells were infected with A/WSN/33 (H1N1, MOI = 5) and treated with 100 nM OA or DMSO as solvent control starting 2 hpi. Transcription of exemplary viral genes was analyzed by quantitative real-time PCR 6 hpi. Data are presented as means + SD of three independent experiments with three biological replicates. Statistical significance was analysed by one-sample t-test.

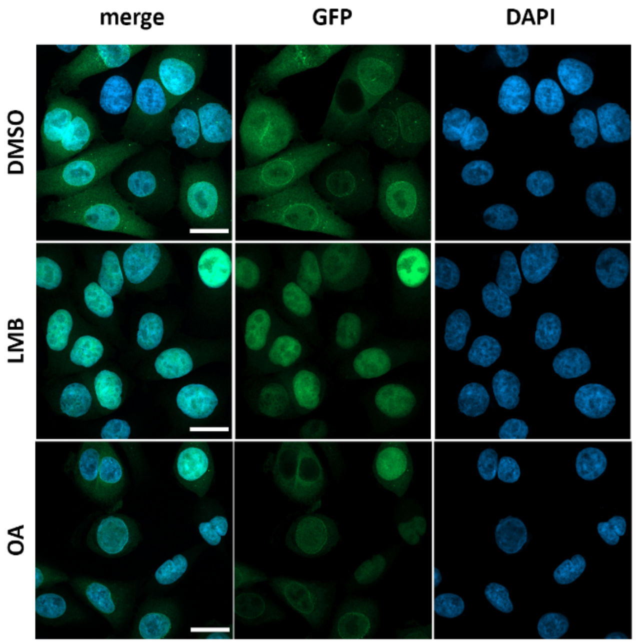

Figure S5: PP2A does not affect the CRM-1-dependent nuclear export. A549 cells stably expressing the GFP biosensor were treated with 5.55 µg/ml leptomycin B (LMB), DMSO or 100 nM Okadaic acid (OA) for 3 h. Cells were fixed in 3.7% formaldehyde and nuclei were stained with DAPI. Scale bar corresponds to 20 µm. Images are representatives of three independent experiments.

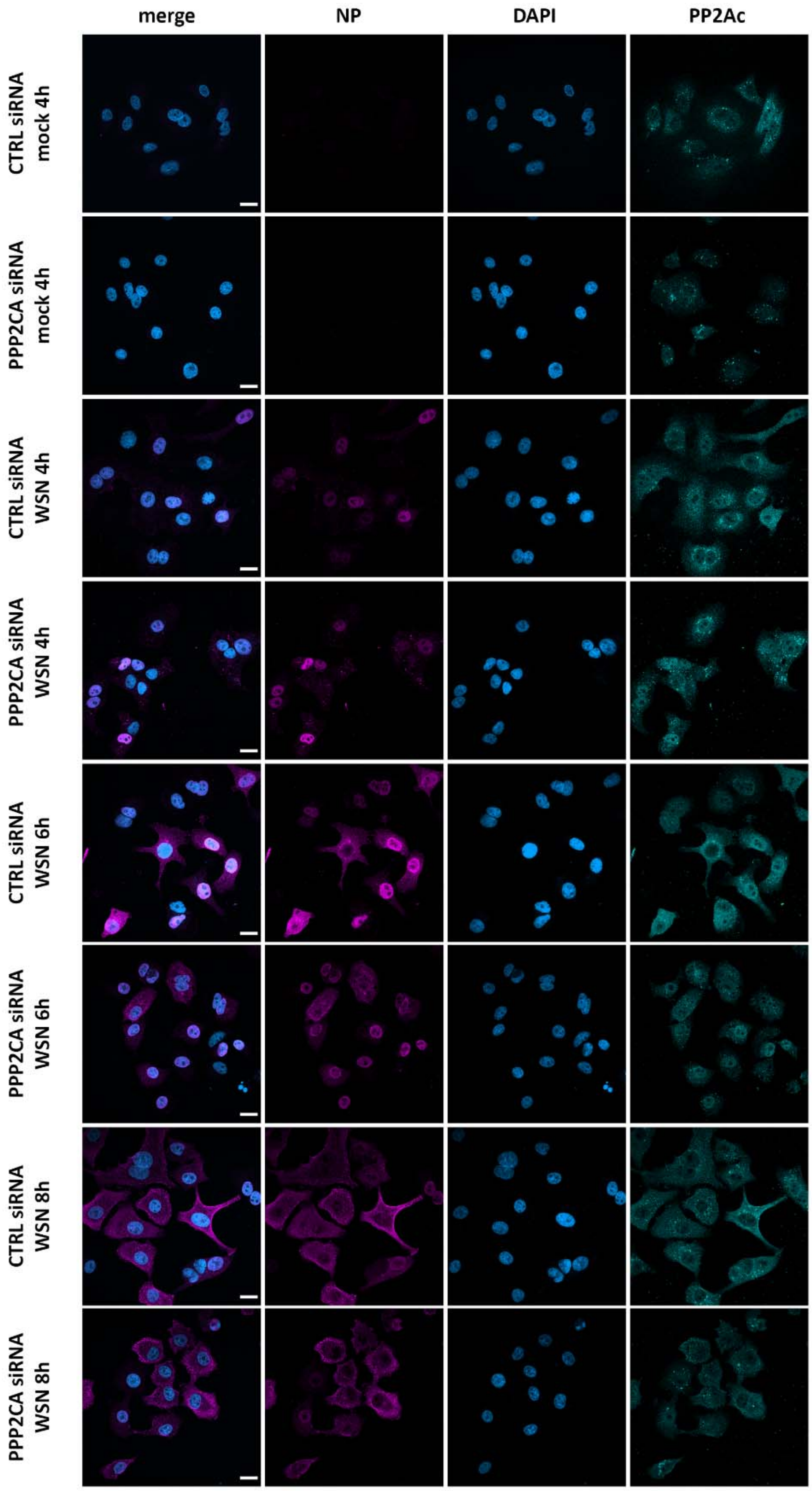

Figure S6: PP2A does not affect trafficking of viral RNP complexes but induces an increased cell death phenotype in IAV-infected cells. A549 cells were infected with influenza A/WSN/33 (WSN, MOI = 5) 48 h after siRNA-mediated KD of PP2Ac. Cells were fixed at the indicated time points in 3.7% formaldehyde and stained with specific antibodies against the IAV nucleoprotein (NP) and PP2Ac. Nuclei were stained with DAPI. Scale bar corresponds to 20 µm. Images are representatives of three independent experiments.

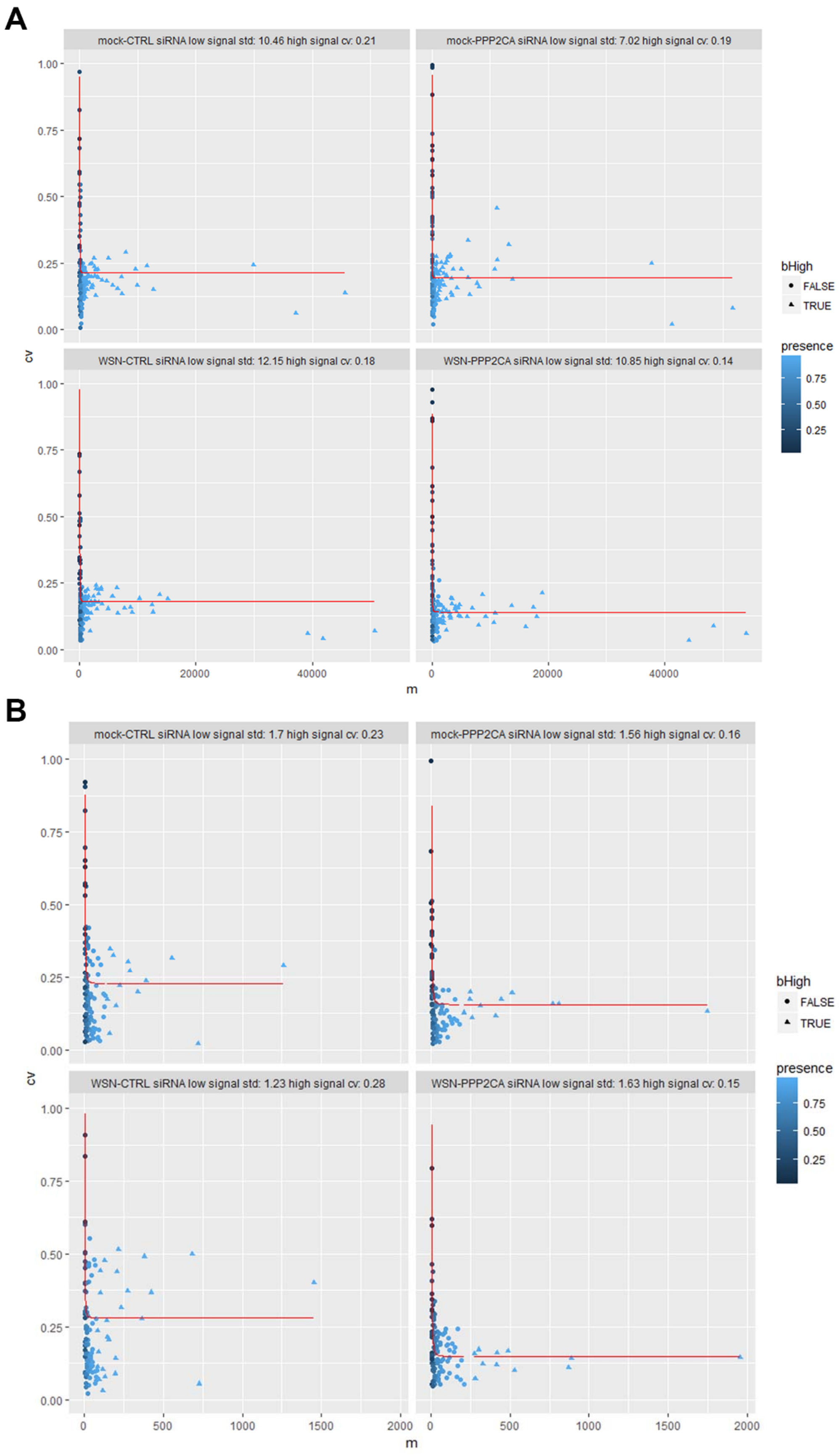

Figure S7: Biological samples used for kinase activity profiling show low variance. A, B) A549 cells were mock-infected or infected with WSN (MOI = 5) 48 h after PP2Ac KD, and the activity of tyrosine kinases (A) and serine/threonine kinases (B) was assessed 8 hpi by chip-based kinase activity profiling (PamGene technology). Depicted are coefficient of variance (CV) plots of three independent experiments.
